# A Challenging Diagnosis of Eosinophilic Cholangitis Requiring Resection of Extrahepatic Bile Ducts

**DOI:** 10.7759/cureus.4925

**Published:** 2019-06-17

**Authors:** Muhammad B Hammami, Levonti Ohanisian, Navneet Kaur, Ahmad Irshad, Angel Sidley

**Affiliations:** 1 Internal Medicine, Charles E. Schmidt College of Medicine, Florida Atlantic University, Boca Raton, USA; 2 Orthopaedic Surgery, Charles E. Schmidt College of Medicine, Florida Atlantic University, Boca Raton, USA; 3 Pediatrics, Charles E. Schmidt College of Medicine, Florida Atlantic University, Boca Raton, USA

**Keywords:** eosinophilic, cholangitis, bile duct

## Abstract

Eosinophilic cholangitis (EC) is a rare benign disease characterized by transmural eosinophilic infiltration of the biliary tree that may result in biliary stricture and obstructive jaundice. The pathophysiology remains unknown but is theorized to involve an allergic mechanism. EC is diagnostically challenging as it may mimic cholangiocarcinoma (CCA) clinically and radiographically and involves a broad differential diagnosis including benign and malignant pathologies. In addition to tumor markers, ultrasonography, contrast-enhanced computed tomography (CT), and magnetic resonance cholangiopancreatography (MRCP), proper evaluation of malignant processes may need biopsy through endoscopic retrograde cholangiopancreatography (ERCP) and surgical exploration. We present the case of a 47-year-old female diagnosed with EC upon exploratory laparotomy.

## Introduction

Eosinophilic cholangitis (EC) is a rare disorder of the biliary tract [1]. Although it is benign in nature, clinically it may mimic cholangiocarcinoma (CCA). It is often difficult to determine the appropriate etiology of patients who present with common hepatic duct or proximal biliary duct obstruction. The differential diagnosis includes malignant causes such as hilar CCA, lymphoma, gallbladder carcinoma and metastatic etiologies as well as benign causes such as autoimmune pancreatitis-associated sclerosing cholangitis, extrahepatic primary sclerosing cholangitis, Mirrizi syndrome, inflammatory strictures secondary to choledocholithiasis, and idiopathic benign focal stricture [1].

Not only can EC mimic CCA in clinical presentation, but is also difficult to differentiate radiographically [2]. Tumor markers may be used to differentiate between benign and malignant causes and diagnosis can be made through imaging of the biliary system using ultrasonography, contrast-enhanced computed tomography (CT), and magnetic resonance cholangiopancreatography (MRCP) [3]. Endoscopic retrograde cholangiopancreatography (ERCP) with biopsy is often needed in order to diagnose malignant pathology [4].

The pathogenesis of EC is poorly understood [5]. Furthermore, there is no strong consensus on diagnostic criteria and treatment [6]. We present the case of a 47-year-old female, diagnosed with EC with histopathologic evidence of eosinophilic infiltration, who required resection of extrahepatic bile ducts with roux-en-y hepaticojejunostomy.

## Case presentation

The patient is a 47-year-old Hispanic American female without significant past medical history who presented with progressive intermittent right upper quadrant (RUQ) abdominal pain of two months duration. The pain was associated with intermittent nausea and an unintentional 10-pound weight loss. She had no personal or family history of gastrointestinal, allergic, or atopic disease. Physical examination was significant for moderate RUQ tenderness. Laboratory testing revealed aspartate aminotransferase (AST) 325 IU/L, alanine aminotransferase (ALT) 130 IU/L, alkaline phosphatase (ALP) 180 IU/L, total bilirubin (TB) 0.7 mg/dl; lipase 38 IU/L; white blood cells (WBC) 11.7 × 103/mm3, with a differential of 55% neutrophils, 14% lymphocytes, and 21% eosinophils. A RUQ ultrasonography revealed evidence of acute cholecystitis without common bile duct (CBD) dilation or evidence of stones. She underwent an uncomplicated laparoscopic cholecystectomy. She improved clinically and was discharged home. Pathology later confirmed eosinophilic cholecystitis.

The following week she presented with recurrent RUQ pain. Laboratory testing revealed AST 631 IU/L; ALT 383 IU/L; ALP 214 IU/L; TB 2.4 mg/dl, lipase 27 IU/L; WBC 15 × 103/mm3, with 53% eosinophils. MRCP revealed mild intrahepatic biliary ductal dilatation with focal soft tissue density of the proximal to middle CBD. Tumor and immunological markers were unremarkable. Given the possibility of a malignant CBD stricture, an ERCP was performed, but without success in passing the guidewire through the stricture. Hence, a percutaneous transhepatic cholangiography (PTC) was inserted for decompression. She improved clinically and was released home. The following week, the abdominal pain recurred. However, it was now associated with fever, chills, and food aversion. Laboratory testing revealed WBC 17.1× 103/mm3, with a differential cell count of 84.5% neutrophils, 4.4% lymphocytes, and 1.4% eosinophils.

**Figure 1:**
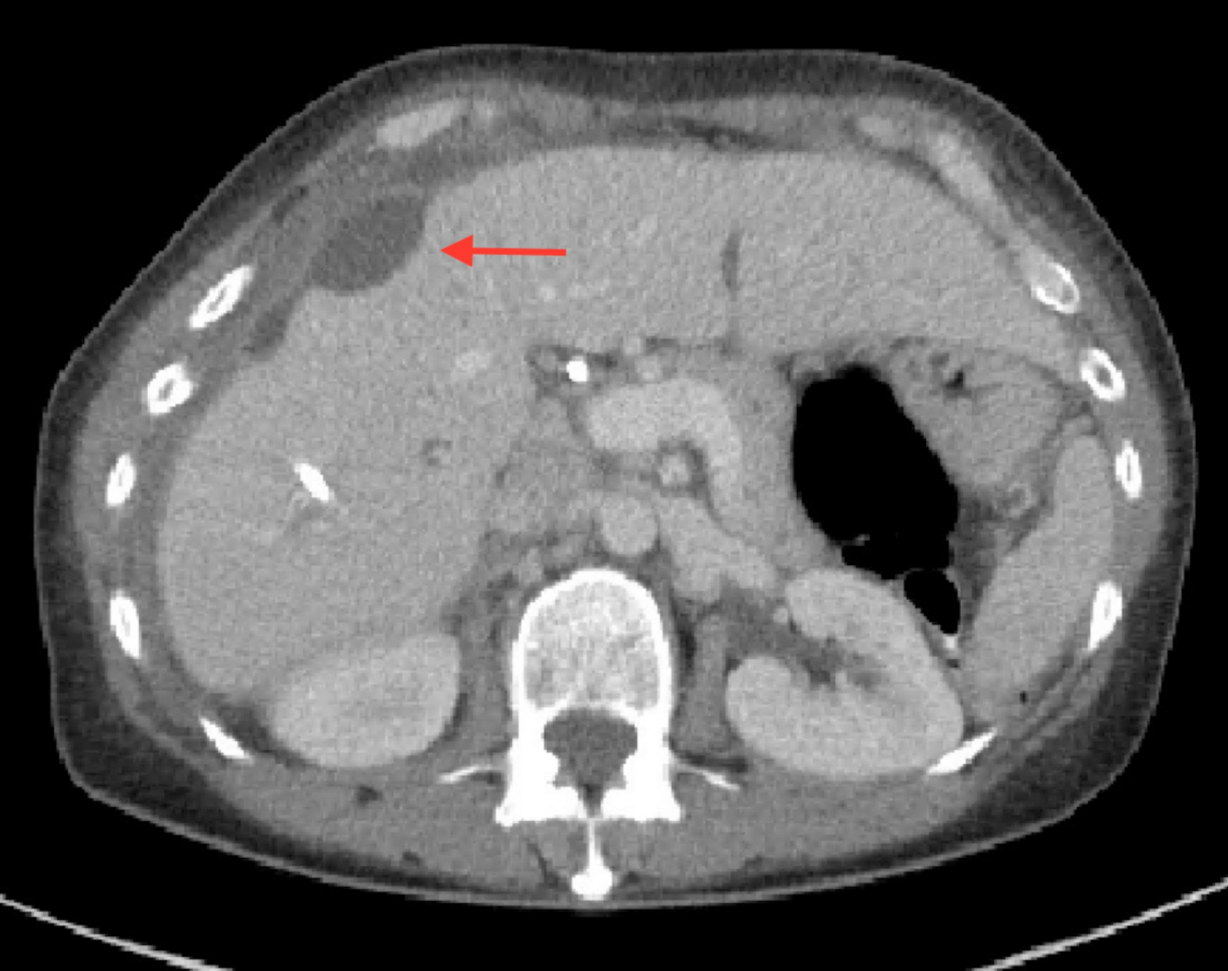
Computed tomography (CT) demonstrating minimal amount of fluid layering adjacent to the liver edge with a small 3-cm focal collection

**Figure 2:**
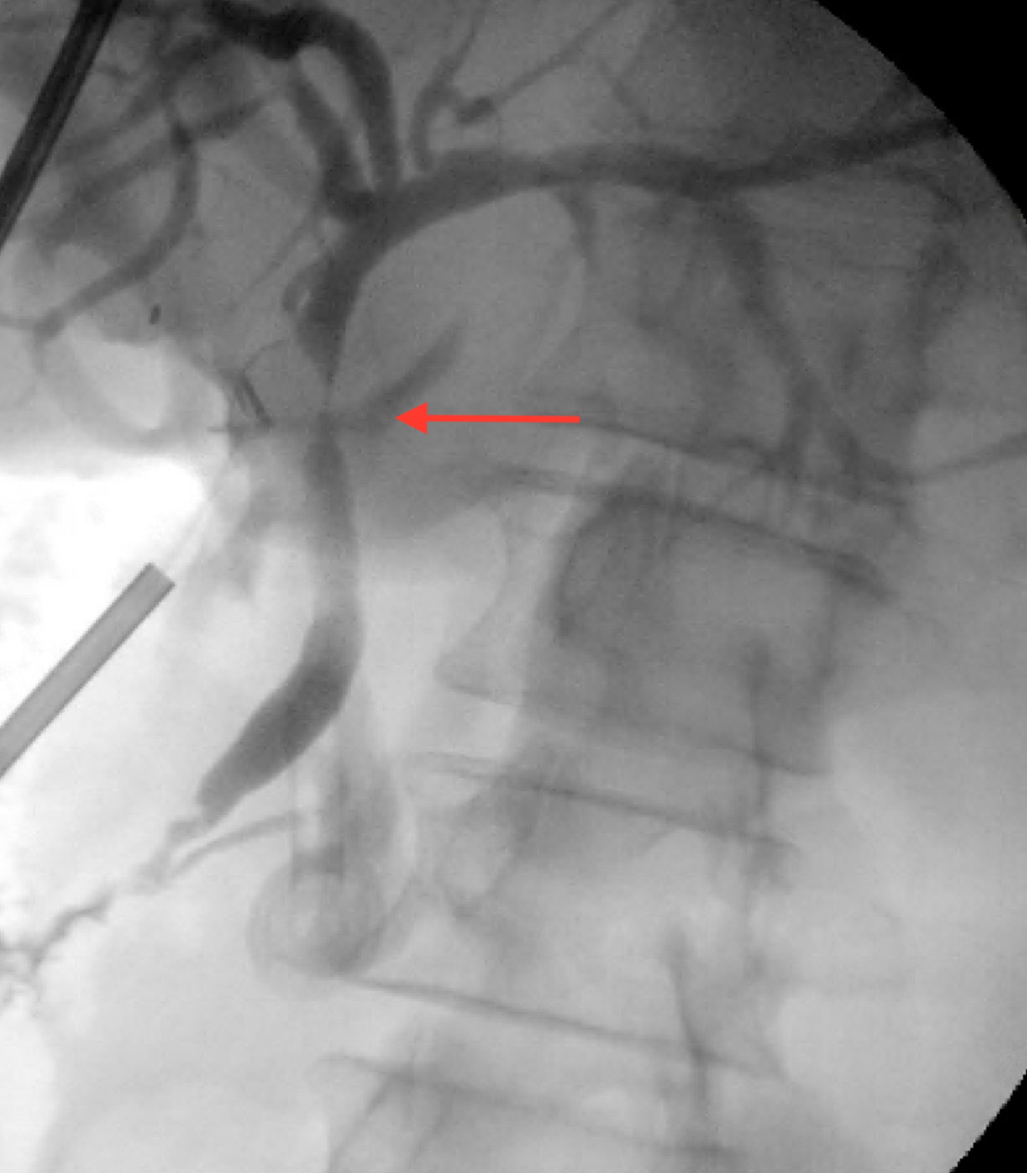
Intraoperative cholangiogram demonstrating proximal short segment common bile duct stenosis

CT scan revealed a 3-cm focal collection adjacent to the liver (Figure 1). She was taken urgently to the operating room for exploratory laparotomy. Intraoperative cholangiogram demonstrated possible proximal short segment common bile duct stenosis (Figure 2). Resection of extrahepatic bile ducts with roux-en-y hepaticojejunostomy was performed along with drainage of the perihepatic abscess and upper abdominal lymphadenectomy. Cultures from the biliary drainage and the perihepatic abscess grew carbapenem-resistant Enterobacteriaceae (CRE) E. coli. Histopathology revealed periductular acute and chronic inflammation, predominantly eosinophilic, with two reactive lymph nodes in areas of increased eosinophils. She was released to a rehabilitation center on post-operative day seven.

## Discussion

EC is a rare benign disease characterized by transmural eosinophilic infiltration of the biliary tree that may result in biliary stricture and obstructive jaundice [3]. The epidemiology involves patients in their fourth or fifth decade of life [5]. Despite being first described by Leegaard et al. in 1985 [7], the pathophysiology of EC is still poorly understood. However, due to the predominance of eosinophils, it is theorized that the pathogenesis involves an atopic mechanism [5]. This mechanism is supported by reports of EC including increased levels of IgE and interleukin 5 [8]. EC presents a formidable diagnostic challenge as it may mimic CCA clinically and radiographically. This adds to the many different diagnoses and may be confused with pathologies such as CCA, lymphoma, gallbladder carcinoma, autoimmune pancreatitis-associated sclerosing cholangitis, extrahepatic primary sclerosing cholangitis, Mirrizi syndrome, inflammatory strictures secondary to choledocholithiasis, and idiopathic benign focal stricture [1].

Due to the broad differential diagnosis, it is important to be able to distinguish between benign and malignant causes of stricture. This differentiation may be accomplished through several laboratory findings such as elevated tumor markers, although CA19-9 may be unspecific to benign and malignant etiologies [9-10]. Diagnosis can be made with several imaging modalities including ultrasonography, contrast-enhanced CT, and MRCP [3]. MRCP may be used to assess for biliary duct narrowing, however, often ERCP and percutaneous transhepatic cholangiography (PTC) may be necessary to determine additional irregularities of the common bile duct and intrahepatic ducts as well as a more detailed analysis of the biliary stricture [11-12].

There are no established diagnostic criteria for EC [6]; however, the triad of wall thickening or stenosis of the biliary system, histopathological findings of eosinophilic infiltration, and reversibility of biliary abnormalities without treatment or following steroid treatment has been proposed by Matsumoto et al. [13]. The degree of eosinophil infiltration has not been established either [6], however, has been suggested as a possible indication of the disease process. Furthermore, there has been no expert consensus on the appropriate treatment for EC and current treatment modalities are based on case reports and case-based experiences [6]. In our case [14], the patient had evidence of wall thickening, stenosis of the biliary system, and histopathological evidence of eosinophilic infiltration supporting aspects of the diagnostic criteria proposed by Matsumoto et al. The reversibility of biliary abnormalities was unclear in our case and was difficult to assess, however, it cannot be excluded and should be a point of emphasis for future research.

In addition to tumor markers, ultrasonography, contrast-enhanced CT and MRCP, proper evaluation of malignant processes may need biopsy through ERCP [4]. Lastly, a surgical evaluation may be needed in order to exclude CCA when the diagnosis is unclear [5], as evidenced in our case. Of note, there is no strong consensus regarding workup for patients with cholecystectomy demonstrating eosinophilic infiltration and the risk of underlying EC in these circumstances. Future investigations are needed to target whether further workup is necessary after histologic evidence of eosinophilic infiltration post cholecystectomy is observed.

## Conclusions

Eosinophilic cholangitis is a rare cause of biliary obstruction. The pathophysiology remains unknown and is theorized to involve an allergic mechanism. While a diagnostic trial of steroids can be attempted, surgical intervention with extrahepatic bile duct excision along with roux-en-y hepaticojejunostomy may be needed as histological confirmation of eosinophilia prior to surgery is often not possible. It should be considered in the differential diagnosis of patients with obstructive jaundice in the setting of peripheral eosinophilia. Despite no established diagnostic criteria, the triad of wall thickening or stenosis of the biliary system, histopathological findings of eosinophilic infiltration, and reversibility of biliary abnormalities without treatment or following steroid treatment has been proposed by Matsumoto et al. Although we were unable to assess reversibility of disease, other aspects of the triad were present in our case, and future cases and reviews are needed to strengthen these diagnostic criteria.

## References

[1] Nashed C, Sakpal SV, Shusharina V, Chamberlain RS (2010). Eosinophilic cholangitis and cholangiopathy: a sheep in wolves clothing. HPB Surg.

[2] Rodgers MS, Allen JP, Koea JB, McCall JL (2001). Eosinophilic cholangitis: a case of 'malignant masquerade'. HPB (Oxford).

[3] Hoilat JN, Hoilat GJ, AlQahtani S, Alhussaini HF, Alabbad SI (2018). Atypical presentation of a rare disease: eosinophilic cholangitis posing as a cancer. Am J Case Rep.

[4] Kurland J, Ozden N, Lee S, Pawa R, Sawhney M, Chuttani R, Pleskow DK (2009). Assessment of SPYGLASS direct visualization system for cholangioscopy and pancreatoscopy in 102 consecutive patients. Gastrointest Endosc.

[5] Fragulidis GP, Vezakis AI, Kontis EA (2016). Eosinophilic cholangitis--a challenging diagnosis of benign biliary stricture: a case report. Medicine (Baltimore).

[6] De Roza MA, Lim CH (2017). Eosinophilic cholangitis treatment with budesonide. World J Hepatol.

[7] Butler TW, Feintuch TA, Caine WP Jr (1985). Eosinophilic cholangitis, lymphadenopathy, and peripheral eosinophilia: a case report. Am J Gastroenterol.

[8] Miura F, Asano T, Amano H (2009). Resected case of eosinophilic cholangiopathy presenting with secondary sclerosing cholangitis. World J Gastroenterol.

[9] Lin MS, Huang JX, Yu H (2014). Elevated serum level of carbohydrate antigen 19-9 in benign biliary stricture diseases can reduce its value as a tumor marker. Int J Clin Exp Med.

[10] Mann DV, Edwards R, Ho S, Lau WY, Glazer G (2000). Elevated tumor marker CA19-9: clinical interpretation and influence of obstructive jaundice. Eur J Surg Oncol.

[11] Vauthey JN, Loyer E, Chokshi P, Lahoti S (2003). Case 57: eosinophilic cholangiopathy. Radiology.

[12] Rosch T, Meining A, Fruhmorgen S (2002). A prospective comparison of the diagnostic accuracy of ERCP, MRCP, CT, and EUS in biliary strictures. Gastrointest Endosc.

[13] Matsumoto N, Yokoyama K, Nakai K (2007). A case of eosinophilic cholangitis: imaging findings of contrast-enhanced ultrasonography, cholangioscopy, and intraductal ultrasonography. World J Gastroenterol.

[14] Sohail M, Hammami MB, Charles C (2018). Eosinophilic cholangiopathy mimicking cholangiocarcinoma: case report. Am J Gastroenterol.

